# Tetrazine bioorthogonal chemistry derived *in vivo* imaging

**DOI:** 10.3389/fmolb.2022.1055823

**Published:** 2022-11-16

**Authors:** Gaoxiang Zhao, Zhutie Li, Renshuai Zhang, Liman Zhou, Haibo Zhao, Hongfei Jiang

**Affiliations:** ^1^ The Affiliated Hospital of Qingdao University, Qingdao University, Qingdao, China; ^2^ Cancer Institute, Affiliated Hospital of Qingdao University, Qingdao, China; ^3^ China United Test and Evaluation (Qingdao) Co. Ltd., Qingdao, China; ^4^ Key Laboratory of Chemistry and Engineering of Forest Products, State Ethnic Affairs Commission, Guangxi Key Laboratory of Chemistry and Engineering of Forest Products, Guangxi Collaborative Innovation Center for Chemistry and Engineering of Forest Products, School of Chemistry and Chemical Engineering, Guangxi Minzu University, Nanning, China; ^5^ Department of Sports Medicine, Affiliated Hospital of Qingdao University, Qingdao, China

**Keywords:** bioorthogonal chemistry, tetrazine click chemistry, *in vivo* imaging, tetrazine probe, live image

## Abstract

Bioorthogonal chemistry represents plenty of highly efficient and biocompatible reactions that proceed selectively and rapidly in biological situations without unexpected side reactions towards miscellaneous endogenous functional groups. Arise from the strict demands of physiological reactions, bioorthogonal chemical reactions are natively selective transformations that are rarely found in biological environments. Bioorthogonal chemistry has long been applied to tracking and real-time imaging of biomolecules in their physiological environments. Thereinto, tetrazine bioorthogonal reactions are particularly important and have increasing applications in these fields owing to their unique properties of easily controlled fluorescence or radiation off-on mechanism, which greatly facilitate the tracking of real signals without been disturbed by background. In this mini review, tetrazine bioorthogonal chemistry for *in vivo* imaging applications will be attentively appraised to raise some guidelines for prior tetrazine bioorthogonal chemical studies.

## Introduction

The aspiration to keep track of life processes from the molecular and protein level has resulted in a convergence of chemistry and biology. Under the circumstances, chemical reactions that occur under physiological conditions gain diverse applications in biological sciences. As early as 2000, Bertozzi and coworkers developed the modified Staudinger reaction within cell surface (Saxon and Bertozzi, 2000). This creative study opened up a wholly new field, bioorthogonal chemistry, wherein unnatural partners be able to react rapidly and selectively under the selected physiological situations in a nonintrusive manner. For more than 20 years, bioorthogonal chemistry has evolved as a highly powerful technique to functionalize and image biological molecules including glycans, nucleic acids, lipids, and proteins in biological systems (Lo, 2020). Encouragingly, the Nobel Prize in Chemistry 2022 has been awarded to Carolyn R. Bertozzi, Morten Meldal, and K. Barry Sharpless, for the development of click chemistry and bioorthogonal chemistry.

Key reactions of bioorthogonal chemistry include Staudinger ligation or native chemical ligation, strain-promoted [3 + 2] cycloaddition reactions, copper-mediated azide—alkyne cycloaddition, metal-mediated coupling reactions, tetrazine ligation, oxime and hydrazone ligations along with photoinducible bioorthogonal reactions (Scinto et al., 2021). Among those reactions, tetrazine bioorthogonal reactions are widely studied in recently years and represent the most significant toolbox for imaging applications. Reasons underlying this should be the rapid kinetics and high selectivity which ensure highly efficient modification even at extraordinary low concentrations normally proceed *in vivo* (Wu and Devaraj, 2018). Tetrazine bioorthogonal reaction refers to inverse electrondemand Diels—Alder chemical reaction between tetrazine derivatives and various dienophiles. This technique was reported by two groups in 2008 independently (Blackman et al., 2008; Devaraj et al., 2008). With continuous efforts, TBC has been applied to numerous chemical biology areas (Oliveira et al., 2017) including biological functionalization and imaging of interested biomolecules (Wu and Devaraj, 2018), metabolic probes development (Rieder and Luedtke, 2014; Agarwal et al., 2015), and bioorthogonal chemistry driven nanotechnologies (Jiang et al., 2021b; Chen et al., 2021).

To facilitate the study of biological functions of biomolecules in their target natural environment, it is inevitable to elaborate highly selective labeling tactics that enable tracking and imaging of the specific biomolecules *in vivo*. The imaging of target biomolecules in selected intrinsically environments may offer a great deal of highly important knowledge on the subcellular localization, changes in expression resulting from diverse stimuli, and interactions with pathogens and neighboring cells (Meineke et al., 2021; Bian et al., 2022). Though constructed genetic fusions bearing fluorescent proteins can regard as a strategy, however, this manipulation may interfere the structure and function of the proteins or glycans, and moreover, lipids cannot be modified with such techniques. Classical bioconjugation approaches can represent alternative strategies but these techniques are mostly confined to *in vitro* manipulations together with low complexity levels (Jiang et al., 2022a; Jiang et al., 2022b). Since bioorthogonal chemistry development, this tactic quickly turns into primary choice for imaging of the specific biomolecules *in vivo*.

Bioorthogonal chemistry-based methodologies for *in vivo* imaging rely on the installation of chemical anchor onto the specific biomolecules, which will further react with the bioorthogonal reagents. In this strategy, a precursor functionalized with a bioorthogonal reaction group is delivered to the target biomolecules in selected cell or organism through metabolic, enzymatic, chemical or nanotechnological approaches. Subsequently, those target biomolecules can be shaped by covalent ligation with probe molecules embodying a complemental bioorthogonal reaction group. Among all the bioorthogonal chemical approaches, TBC has been wildly applied in various areas and represent the mostly significant and efficient tactic in this area (Wu and Devaraj, 2018). Reasons that bioorthogonal chemistry is suitable choice include 1) no cross-reactivity or interference with intrinsically occurring functionalities take place; 2) it show moderate to high reactivity under physiological situations; and 3) those employed reactions do not induce severe toxicity (Borrmann and van Hest, 2014). In this mini review, a selection of state-of-the-art TBC for animal *in vivo* imaging and their target promising application in biological situations is summarized. We will appraise in detail the newly developed methodologies in the recent years. This review also endeavors to demonstrate the detailed mechanisms to help researchers inspire the development of future tetrazine bioorthogonal methodologies.

## Building blocks applied in TBC

Most of the biologically utilized tetrazines are 1,2,4,5-tetrazine derivatives, the molecules that are developed long ago, whose first synthetic route was reported 110 years ago by Hofmann and coworkers (Hofmann and Ehrhart, 1912). Over the years, very few groups that turned interest toward the 1,2,4,5-tetrazine preparation. Driven by TBC, the preparation of tailored molecules with broad applications in Diels-Alder cycloaddition reactions to make new pyridazines is thriving in recent decades. Devaraj group has made a lot of contributions to 1,2,4,5-tetrazine preparation, modification and biological applications (Wu et al., 2016; Wu and Devaraj, 2018). They firstly developed lewis acid-metal complex promoted one-pot synthesis method for the preparation of some benzyl-1,2,4,5-tetrazines (Yang et al., 2012a). To continue their efforts to avoid the limitations of metal catalyzed *de novo* tetrazine preparation, Devaraj group sought to utilize Heck coupling to prepare tetrazine building blocks for TBC applications which could be linked to selected functional molecules efficiently *via* very mild reaction condition. This one-pot elimination-Heck cascade coupling methodology has contributed a lot to functionalized 1,2,4,5-tetrazine bioorthogonal probe preparation (Wu et al., 2014).

**FIGURE 1:**
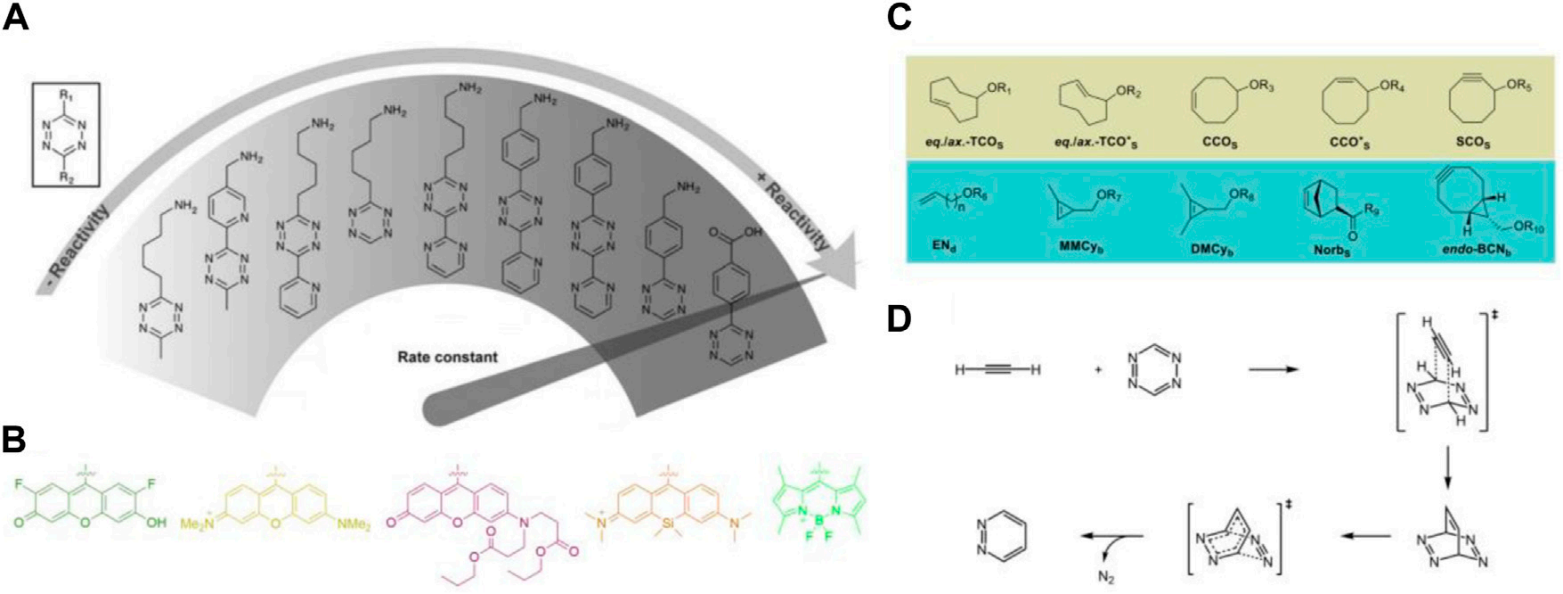
General information of the building blocks applied in TBC. **(A)** Reactivity of selected tetrazines in Diels-Alder cycloaddition. **(B)** Selected fluoresce reporter on tetrazine motif with off/on mechanism. **(C)** Commonly utilized dienophiles in Diels-Alder cycloaddition. **(D)** Mechanisms of the Diels-Alder cycloaddition.

Tetrazine bioorthogonal reactions generally include Diels-Alder cycloaddition with dienophiles and [4 + 1] cycloaddition with isonitriles. For *in vivo* applications, Diels-Alder cycloaddition is much more popular attributed to its fast and tunable kinetics and can be used *in vivo* with nanomolar concentrations (Oliveira et al., 2017). Present endeavors of researchers mainly focus on preparing novel 1,2,4,5-tetrazine derivatives (Figure 1A) with useful functional groups (Figure 1B) as well as screening of suitable dienophile substrates (Figure 1C). Commonly used dienophiles include the axially and equatorially linked equatorial and *trans*-cyclooct-2-ene isomers (*eq*.-TCO*_S_ and *ax.*-TCO*_a-d_) (Hoffmann et al., 2015), *endo*-bicyclo [6.1.0]non-4-yne (*endo*-BCN_a-c_) (Dommerholt et al., 2010), mono- and dimethylcyclopropene derivatives (MMCy_a-d_ along with DMCy_a-d_) with selected linear amino acids of different length *via* a carbamate (Yang et al., 2012b), cycloct-2-yn-1-ol (SCO_S_) derivatives (Plass et al., 2011), 2*Z* or 4*Z* isomers of *cis*-cyclooctenol (CCO_S_) (Zhou et al., 2020) and norbornene substrate (Norb_S_) (Best et al., 2015). Incorporating the most frequently used 1,2,4,5-tetrazine and dienophile moieties from literature, the entire reactivity (*k*_*2*_) range from 10^−2^ m^−1^s^−1^ to 10^5^ m^−1^s^−1^ (Lang and Chin, 2014).

Investigation into the optimization of 1,2,4,5-tetrazine motifs to optimize and expand their biological applications, researchers have organized various 1,2,4,5-tetrazine derivatives with functional groups at the C-3 or C-6 position. Several research groups, including Devaraj group (Devaraj, 2018; Wu and Devaraj, 2018), Prescher group (Row and Prescher, 2018) and Wu group (Mao et al., 2021) have made large and significant contributions to developing new routes to 1,2,4,5-tetrazines and expanding the range of valuable functional groups, with specific focus on the development of functional groups with off-on mechanisms (Figure 1B). Derivatization approaches of tetrazine require the functionalization of electron-donating groups to balance the electrondeficient skeleton or electron-withdrawing groups to improve the reaction kinetics (Maggi et al., 2016). Under the circumstance, various of alkyl and aryl tetrazines have been organized and prepared for selected purpose. The 1,2,4,5-tetrazine motifs with different dyes at C-3 or C-6 position can act as reporters *in vivo*, while the substituted groups of carboxyl, amino or biotin are reactive sites and can be further modified to fulfill the target biological application requirements. Mechanisms underlying the tetrazine Diels-Alder cycloaddition has been presented in Figure 1D, in which the 1,2,4,5-tetrazine and dienophile (ethyne as example) comprise of the cycloaddition reaction to give tetraazabarrelene as an intermediate, consequently, the elimination of N_2_ will form the final product pyridazine.

## Glycan-based TBC for *in vivo* imaging

Along with the rapid development of genomics and proteomics, glycomic analysis is gaining increasing attention in biological and biomedical studies of glycans. Glycans on cell surface or protein mediate numerous of critical biological functions, such as viral along and bacterial infection, inflammation, angiocardiopathy, embryogenesis and cancer (Jiang et al., 2021a; Tabang et al., 2021). On account of intricate structures as well as non-template-driven preparation, the glycans are usually hard to interrogate and manipulate compared with the other biomolecules, for instance, oligonucleotides and proteins. What’s more, the physiological significance of many glycans has induced the requirement for *in vivo* imaging technologies in their native physiological conditions. Antibodies and lectins targeting selected glycan epitopes can detect glycans *in vitro* but are ill-suited for *in vivo* imaging owing to their low affinity for glycans and poor tissue penetrance (Laughlin and Bertozzi, 2009).

**FIGURE 2:**
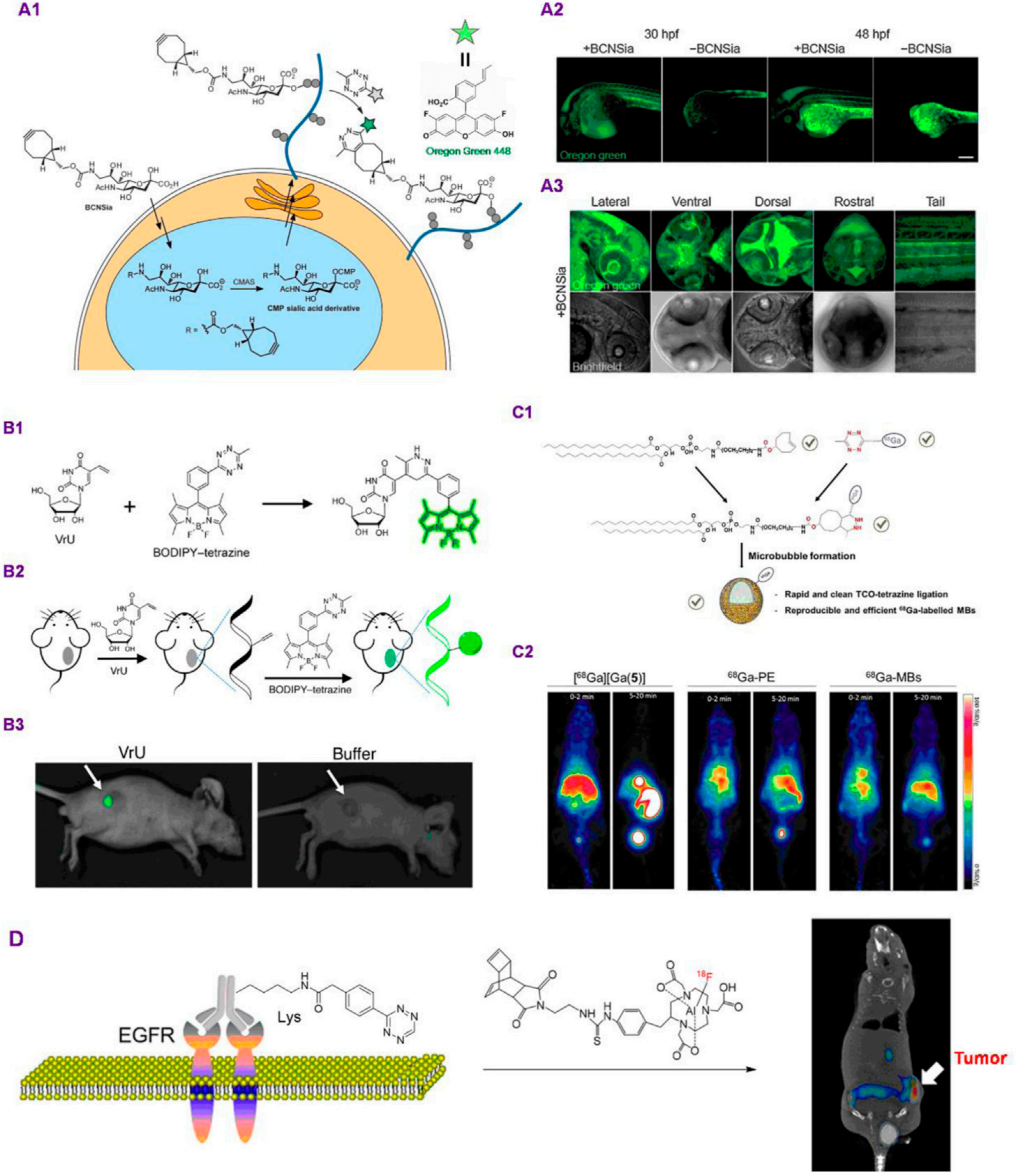
Applications of TBC for animal *in vivo* imaging. **(A)** Glycan-TBC for *in vivo* imaging. **(A1)**: Metabolic route for functionalization of BCNSia into cell-surface glycan analogs and its functionalization with tetrazine reporter; **(A2)**: projection images of 30 or 48 hpf embryos deal with BCNSia or vehicle; A3: projection images (20×) of 48 hpf embryos from a number of viewpoints (Copyright 2015 Wiley-VCH). **(B)** TBC for nucleic acids *in vivo* imaging. **(B1–B2)**: Schematic of Diels−Alder reaction between BODIPY−tetrazine with VrU acted as the light-up reporter method; **(B3)**: *in vivo* imagination of mice from buffer along with VrU groups, tumor tissues are labelled with arrows (Copyright 2019 American Chemical Society). **(C)** Lipid-TBC for *in vivo* imaging. **(C1)**: Development of bioorthogonal building blocks for “click” ligation for the preparation of ^68^Ga-labelled MBs. **(C2)**: PET imagination of Balb/c nude mice after intravenous injection of ^68^Ga (left), ^68^Ga-labelled PE (middle) and, ^68^Ga-labelled MBs (right) at different time intervals (Copyright 2019 Royal Society of Chemistry). **(D)** Pretargeted immuno-PET rely on TPC for imaging EGFR positive colorectal cancer (Copyright 2018 American Chemical Society).

As an alternative to these affinity-based approaches, using biorthogonal chemical reporter strategy to manipulate and image glycans is very promising. An exciting strategy by Bertozzi group incorporates the metabolic incorporation of functionalized-cyclooctyne- sialic acid analogs with ligation reaction of fluorogenically tetrazine derivatives, fullfilling the imaging of glycans and glycoconjugates inside living zebrafish embryos (Figure 2A). It is worth to mention that normal technique to the system-wide modification of cell-surface glycan analogs is to combine the selected metabolic labeling reagent and the target imaging reporter into a developing embryo through injection. Nevertheless, the excess probe can be visualized inside the organism, resulting in strong background fluorescence that would mask signals from glycan analogs of interest. The issue was obviated by the use of a 1,2,4,5-tetrazine-based fluorogenic probe whose fluorescence signal was activated in the system of the target ligation reaction with cyclooctyne motif, which were initiated by Devaraj and coworkers and exhibit up to 400-fold fluorescence turn-on properties (Agarwal et al., 2015). Ahead of bioorthogonal ligation process, metabolic incorporation of the target bicyclononyne-functionalized sialic acid (BCNSia) was carried out. Following was the reaction with a fluorescence turn-off Oregon Green 488 functionalized tetrazine to enable the systemic visualization of sialylation during the zebrafish embryogenesis progress (Figure 2A1). The studies suggested that sialylation was plentiful within a few structures, the situations were investigated in detail in embryos injected at 30 to 48 hpf (hours post fertilization) with tetrazine probe (Figure 2A2). The BCNSia dependence of modification was especially striking in higher-magnification imaginations of its head acquired from the lateral, rostral ventral, and dorsal views (Figure 2A3).

Antibody-based bioorthogonal pretargeting strategy for *in vivo* nuclear imaging has been developed by Zeglis group. The work combined bioorthogonal pretargeting strategy and site-selective bioconjugation tactic to formulate a pretargeted positron emission tomography (PET) imaging strategy depending on the fast and bioorthogonal inverse electron demand Diels−Alder cycloaddition among the ^64^Cu-modified tetrazine ligand (^64^CuTz-SarAr) and the site-selectively functionalized *trans*-cyclooctene- huA33 immunoconjugate (PEG-TCO- huA33). Eventually, mice were incorporated with PEG-TCO- huA33 through tail vein injection followed by accumulation intervals of about 24–48 h Cu-Tz-SarAr. In addition, PET imaging and biodistribution investigations unveiled that the technique very well delineates tumor tissues as early as 1 h post-injection, formulatingimages with outstanding contrast as well as distinct tumor-to-background activity concentration ratios (Cook et al., 2016; Sarrett et al., 2021). This strategy has a similarity with Brindle’s work whose tetrazine reporter was fluorescent dye functionalized tetrazine derivatives (Neves et al., 2013).

## TBC for nucleic acids *in vivo* imaging

Accurate assessment of the distinctness in tumor associatednucleic acid expression levels presents meaningful information for tumor.

Expression levels presents meaningful information for tumor treatment and diagnosis. Up to now, some strategies, such as genetically encoded sensors, molecular beacons, together with spherical nucleic acids are developed and have been applied to detect nucleic acids in living cells (Gao et al., 2021; Zhao et al., 2022). These strategies mainly rely on the hybridization with the selected nucleic acid and deliver the corresponding inadequate signal output, which would disturb the detection of cellular nucleic acid with very low copy numbers. Eventually, the DNA cascade circuits based on toehold-depended strand displacement to fulfil highly potent nonenzymatic signal amplification have been developed for sensitive RNA imaging in live cells (Wei et al., 2020; Wu et al., 2020). Despite these significant progresses, the conventional cascades with selected fluorophores and quenchers might be degraded by the endogenous nucleases within cellular environments and causing high background signals or even false the real positive results (Wu et al., 2015; George and Srivatsan, 2020).

Nucleic acid-templated bioorthogonal reactions, mainly the tetrazine ligation, have been regared as a very promising method for the monitoring or imaging of nucleic acids *in vivo*. 5-Ethynyluridine (EU) and 5-ethynyl-2′-deoxyuridine (EdU) derivatives were utilized to monitor RNA and DNA, respectively, in live cells. Liu and coworkers developed a nucleoside analog 5-vinyluridine (VrU) for modification of during cell division along with for tumor tissue imaging in the living mice (Liu et al., 2019). In this work, functional nucleosides bearing a VrU was metabolically incorporated to RNA inside cell, which can be applied to the imagination of RNA using a 1,2,4,5-tetrazine mediated bioorthogonal Diels−Alder reaction (Figures 2B1–B2). They further spreaded this tactic to observation of RNA together with DNA behaviors in a group of primary stages of the cell division as well as for tumor tissue imagination in living mice (Figure 2B3). In addition, the study applied the VrU and EdU to observe RNA together with DNA simultaneously at single-cell resolution. Instead of using 1,2,4,5-tetrazine probe as the reporter, Zhang group designed several coumarin-fused 1,2,3,4-tetrazoles for the realization of “photoclick” labeling and imaging of DNA *in vivo*. They demonstrated very rapid (up to 19.5 M^−1^ s^−1^) fluorogenic imagination of DNA *in vivo* depending on rationally devised coumarin-modified tetrazole derivatives under the UV LED photoirradiation. Based on a water-soluble and nuclear-selective coumarin-fused tetrazole analog (CTz-SO_3_), the metabolically organized DNA in live cells was efficiently modified and visualized through “photoclick” reaction and without fixation. Subsequently, the photoclickable CTz-SO_3_ building block enabled spatially regulated imagination of DNA in the live zebrafish (Wu et al., 2019).

## Lipid-based TBC for *in vivo* imaging

Lipids are significant and valuable building blocks of cells, they play critical roles in energy storage, membrane formation, and signaling. Lipids are greatly diverse in chemical structure, furthermore, their distribution varies with different organisms. Despite the vital relevance and intrinsic *in vivo* functions, lipids are still less investigated than equally essential biomolecules. One important reason should be the relative shortage of strategies to interrogate their manipulation as well as visualize them (Williams and Grant, 2019). Furthermore, it is quite difficult to accommodate all species of them with normal strategies of extraction, purification, and analysis owing to the high structural diversity of lipid families. Over the last decades, probes target lipid have become powerful tools in synthetic biology and new bioorthogonal tactics have been demonstrated for imaging lipids in their physiological conditions (Flores et al., 2020).

Lipids are the basic building blocks for microbubbles (MBs), the contrast agents that play a critical role in fields of anatomical and molecular imaging and can be utilized pre-clinically and clinically. In those applications, MBs act as driving force which can prevent drawbacks inherent to the already developed imaging modalities (Mewis and Archibald, 2010; Abou-Elkacem et al., 2015; Wang et al., 2021). Nevertheless, its inability to supply whole-body imagination can severely obstruct the exploitation of novel MB formulations. Long group described a rapid and highly efficient strategy for achieving the labeling of MBs by using ^68^Ga (Hernandez-Gil et al., 2019). The approach produced ^68^Ga-labeled MBs in excellent isolated yields by means of the bioorthogonal inverse-electron-demand Diel–Alder cycloaddition between cyclooctene-functionalized phospholipid and the novel tetrazine-fused CC-HBED chelator. Bioorthogonal reaction of TCO and phospholipids was simple, efficient and reproducible. In addition, the novel CC-HBED-tetrazine chelator supplied quite efficient ^68^Ga-labeling with high yields, this method produced reproducible preparation of ^68^Ga-MBs under mild and controllable conditions (Figure 2C1). In addition, this strategy offered real-time imaging along with the ability of easily customising tunable phospholipid-based formulations. Furthermore, they confirmed that the corresponding ^68^Ga-MBs permit non-invasive investigation of the *in vivo* whole-body distribution of MBs in mice (Figure 2C2).

As promising vehicles for controlled release of cytotoxins and drugs, liposomes have a very long-standing history in clinical practice and medical study. Moreover, liposomes possessseveral advantageous capabilities in molecular imaging applications, such as improved stability and the capability to be modified with radioisotopes, along with paramagnetic or fluorescent contrast analogs. Emmetiere and coworkers applied bioorthogonal liposomes for *in vivo* imaging study. They coated radiolabeled liposomes with trans-cyclooctene and pretargeting with a tetrazine coupled to selected polypeptide, which were capable to improve the retention of the liposomes and combine them with tumor in live animals. Subsequently, the bioorthogonally driven tumor-targeting of liposomes through *in vivo* click reaction was very attractive and was able to be explored for more sensitive delivery of radiodiagnostics and radiotherapeutic (Emmetiere et al., 2013).

## Protein-based TBC for *in vivo* imaging

Uncovering the secret of protein structure and function is essential for understanding various biology processes, but it remains very challenging owing to the high intricacy of protein networks. In addition, the daunting assignment of elucidating these complicated interconnections demands the concerted application of strategies derived from various disciplines. Site-selective protein modification with functional agents, for instance, spin probes, fluorophors, and affinity tags has considerably assisted both *in vitro* and *in vivo* investigations of the structure and function of protein. Bioorthogonal reactions can facilitate the regioselective functionalization of selective chemical agents to proteins, which are extremely promising techniques for site-specific protein labeling (Chen et al., 2011; Bird et al., 2021). Thereinto, antibody based TBC is widely studied and possesses applications in imaging and therapeutic fields. As illustrated in Figure 2D. Pretargeted immuno-PET imagination technique rely on the reaction between ^18^F-fused dienophiles and tetrazine building blocks of two EGFR-targeting monoclonal antibodies panitumumab and cetuximab was developed (Shi et al., 2018). Firstly, lysine residue of EGFR-specific monoclonal antibodies (panitumumab or cetuximab) was labeled with tetrazine. The monoclonal antibodies−tetrazine motifs were subsequently utilized to conect with the positron emitter modified dienophiles *in vivo*. Immuno-PET imaging as well as biodistribution investigations revealed a fast hepatobiliary along with renal excretion and a subsequent low background signal of the probe, leading to a high quelity along with unobstructed imagination of EGFR expression in living mice (Figure 2D). Alternatively, a^18^F-labeled tetrazine was designed as a bioorthogonal reporter to react with a TCO handle on tumor targeting antibody (Battisti et al., 2021). This ^18^F-labeled tetrazine has favorable target-to-background ratios and good pharmacokinetics in its *in vivo* pretargeted PET imaging investigation. The probe has been considered to have the capacity to be clinically applied for *in vivo* pretargeted PET imagination by the authors. Need to mention that their results were preliminary studies and require detailed investigation using more complex models (e.g., PDX or orthotopic).

## Perspectives

Bioorthogonal chemistry pursuit the selective reactions in harsh environments. The strict demand of the technique for *in vivo* applications has forced researchist to develop unconventional handles into the *in vivo*, which usually represent complicated prior task. Fortunately, several clinical or pre-clinical drugs relying on bioorthogonal chemistry (e.g., TRPH-222, STRO-002, ADCT-601 et al.) are ongoing to explore drugabilities of this technique (Peplow, 2019).

The tetrazine bioorthogonal reactions summarized in this mini-review and related *in vivo* imaging applications have proven to be potent tools. Thus, tetrazine mediated Diels-Alder cycloaddition can successfully be utilized for site-specific modification of glycans, nuclear acids, lipids and proteins under physiological conditions, which can facilitate the tracking and imaging of those important biomolecules in their physiological conditions. Depending on these tactics the orientation of labeled biomolecules can be controlled while retaining their structures coupled with activities, which greatly assists the study of the biological functions of in their target natural environment. Expanding the repertoire of TBC has supplied new opportunities for chemo- and site-specific functionalization of biomolecules and the related biological applications. Nevertheless, despite the achievement in developing such strategies in past years, the demand for novel tetrazine bioorthogonal reactions with greatly improved selectivities, kinetics, the ability to be operated at lower concentrations, and the complementary with existing bioconjugation techniques remains high. Another consideration is the accessibility of the reagents used for the tetrazine bioorthogonal reactions. Many of present tetrazine and dienophile building blocks are prepared with multiple steps coupled with the moderate to low yields. Tetrazine and dienophile motifs that are commercially available or can be prepared in simple synthetic steps from commercially available building blocks will have a great vogue. Continued efforts are poised in this promising field.
